# Carboxyl-terminal domain of MUC16 imparts tumorigenic and metastatic functions through nuclear translocation of JAK2 to pancreatic cancer cells

**DOI:** 10.18632/oncotarget.3308

**Published:** 2015-01-21

**Authors:** Srustidhar Das, Satyanarayana Rachagani, Maria P. Torres-Gonzalez, Imayavaramban Lakshmanan, Prabin D. Majhi, Lynette M. Smith, Kay-Uwe Wagner, Surinder K. Batra

**Affiliations:** ^1^ Department of Biochemistry and Molecular Biology, University of Nebraska Medical Center, Omaha, NE, USA; ^2^ Department of Biostatistics, University of Nebraska Medical Center, Omaha, NE, USA; ^3^ Department of Pathology, University of Nebraska Medical Center, Omaha, NE, USA; ^4^ Buffett Cancer Center, Eppley Institute for Research in Cancer and Allied Diseases, University of Nebraska Medical Center, Omaha, NE, USA

**Keywords:** Mucin 16 (MUC16), CA125, pancreatic cancer, JAK2, cancer stem cells

## Abstract

MUC16 (CA125) is a type-I transmembrane glycoprotein that is up-regulated in multiple cancers including pancreatic cancer (PC). However, the existence and role of carboxyl-terminal MUC16 generated following its cleavage in PC is unknown. Our previous study using a systematic dual-epitope tagged domain deletion approach of carboxyl-terminal MUC16 has demonstrated the generation of a 17-kDa cleaved MUC16 (MUC16-Cter). Here, we demonstrate the functional significance of MUC16-Cter in PC using the dual-epitope tagged version (N-terminal FLAG- and C-terminal HA-tag) of 114 carboxyl-terminal residues of MUC16 (F114HA). *In vitro* analyses using F114HA transfected MiaPaCa-2 and T3M4 cells showed enhanced proliferation, motility and increased accumulation of cells in the G2/M phase with apoptosis resistance, a feature associated with cancer stem cells (CSCs). This was supported by enrichment of ALDH^+^ CSCs along with enhanced drug-resistance. Mechanistically, we demonstrate a novel function of MUC16-Cter that promotes nuclear translocation of JAK2 resulting in phosphorylation of Histone-3 up-regulating stemness-specific genes *LMO2* and *NANOG.* Jak2 dependence was demonstrated using Jak2^+/+^ and Jak2^−/−^ cells. Using eGFP-Luciferase labeled cells, we demonstrate enhanced tumorigenic and metastatic potential of MUC16-Cter *in vivo*. Taken together, we demonstrate that MUC16-Cter mediated enrichment of CSCs is partly responsible for tumorigenic, metastatic and drug-resistant properties of PC cells.

## INTRODUCTION

Pancreatic cancer (PC) confers a near 100% mortality, a dismal 5-year survival rate of 5% and a median survival of 5-8 months [1]. The bleak prognosis associated with PC is primarily due to the advanced (metastatic) stage at the time of clinical diagnosis and the refractory nature to conventional chemo and radiotherapy [1]. Recent studies using lineage tracing in genetically engineered mouse models and mathematical modeling using patient datasets have proposed early dissemination of PC cells to establish the metastatic disease [2,3]. These early-disseminated cells called circulatory pancreatic cells (CPCs) are of mesenchymal type and possess the characteristics of cancer stem cells (CSCs) [2]. A number of recent studies have demonstrated the significance of CSC in metastasis, chemo resistance and disease recurrence in various cancers including PC [4-6]. Therefore, our understanding of the mechanism(s) of CSC enrichment and maintenance will be critical in devising successful therapeutic strategies against the lethal PC.

Membrane bound mucins represent a special class of type-I transmembrane proteins capable of sensing the extracellular milieu with the large extended extracellular domain and participate in cellular signaling with the carboxyl-terminal fragment that is hypothesized to be released following proteolytic cleavage [7-10]. Interestingly, these transmembrane mucins are proposed to possess the ability to cleave (autoproteolytic and/or by proteases) off the C-terminal region from the rest of the protein primarily at the membrane proximal SEA (Sperm protein, Enterokinase and Agrin) domain with the exception of MUC4, which does not have one [11-13]. In recent years a lot of interest has been generated in the involvement of mucin cytoplasmic tail, particularly MUC1-Cter, in cellular signaling rather than just a structural component. Although MUC1 is the best characterized transmembrane mucin with respect to cleavage and oncogenic signaling [7,12,14], targeting MUC1 has not been quite successful. Multiple mucins are expressed by the same patients; therefore, it is important to understand the common and unique features of these predominant mucins for devising successful mucin based therapeutic targeting.

MUC16, the largest known transmembrane mucin, is encoded by 22,152 amino acids comprised of a heavily glycosylated N-terminal region encompassing the tandem repeat region with > 60 repeats of ~156 amino acids each in which most of the SEA domains are interspersed, a transmembrane domain and a cytoplasmic tail domain (CTD) of 32 amino acids [9,10,15]. It acts as a precursor for CA125, most widely used serum biomarker for ovarian cancer (OC), that are located as the repetitive peptide epitopes in the large glycosylated N-terminal region of MUC16, therefore, is of high clinical importance [16]. However, our understanding of the biological role of MUC16, particularly during oncogenesis, is very limited. Recent studies show that in addition to OC, MUC16 is expressed in multiple cancer types and is associated with poor prognosis [17,18]. Further, MUC16 knockdown studies in breast, ovarian and PC cells associated it in imparting protumorigenic, prometaststic, chemo resistant and anti-apoptotic properties to cancer cells [19-23]. In addition, ectopic expression of different lengths of carboxyl-terminal MUC16 (283 and 413 amino acids) in ovarian, colon and breast cancer cells resulted in increased metastatic and chemo resistant properties, suggesting it to be critical in mediating the functions of MUC16 [19,20,24]. Our understanding, however, of the existence and generation of a cleaved MUC16 and its role in tumorigenesis is still limited. In another study, we performed in-depth analysis of MUC16 cleavage and have shown its cleavage to be distinct from the previously predicted sites (Das et al., submitted for publication elsewhere). Generation of the cleaved MUC16 in our experimental system is further supported by the endogenous existence of a ~17 kDa MUC16-Cter fragment carried out using an in-house antibody for the CTD of MUC16 in NHBE cells [25].

Janus kinase 2 (JAK2) belongs to a family of nonreceptor cytoplasmic tyrosine kinase implicated in multiple cellular processes primarily mediated by the phosphorylation induced dimerization and nuclear translocation of its target proteins signal transducers and activators of transcription (STATs) [26,27]. Classically, JAK2 has been shown to be active only in the cytoplasm, however, recent studies in hematopoietic cells and embryonic stem cells suggest an unusual nuclear role of JAK2 in up regulating genes such as *LMO2* [28] and *NANOG* [29] implicated in inducing stem cell-like features during carcinogenesis [30-32].

In our previous study, we showed *de novo* expression of MUC16 in the high-grade preneoplastic lesion, primary as well as metastatic PC with metastatic tumors having stronger MUC16 expression compared to the primary tumors from the same patient [33]. In the present study, we report (i) the generation of a 17-kDa cleaved MUC16 (MUC16-Cter) using dual-epitope tagged 114 amino acids of carboxyl-terminal MUC16 in PC cells, (ii) MUC16-Cter mediated enrichment of ALDH^+^ cancer stem-like cells imparts tumorigenic, metastatic and drug resistant properties to PC cells and (iii) MUC16-Cter mediated enrichment of stemness specific genes *LMO2* and *NANOG* is dependent on nuclear JAK2.

## RESULTS

### Expression of dual-tagged 114 amino acids of carboxyl-terminal MUC16 generates a ~17 kDa cleaved MUC16 and imparts proliferative advantage to PC cells

**Figure 1:**
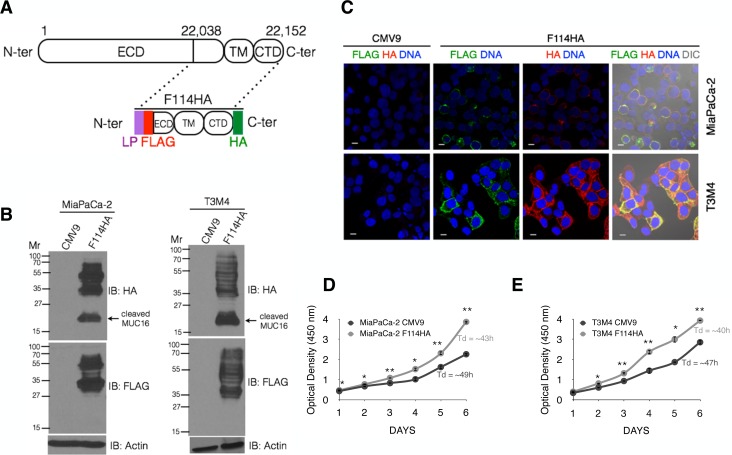
Ectopic expression of 114 amino acids of carboxyl-terminal MUC16 promotes the *in vitro* proliferation of PC cells (A) Schematic representation of full-length and 114 amino acids of carboxyl-terminal MUC16 with N-terminal FLAG and C-terminal HA-tag (F114HA) cloned into the p3X-FLAG-CMV9 vector (CMV9) with a preprotyrpsin leader peptide (LP). (B) MiaPaCa-2 (MUC16-non-expressing) and T3M4 (MUC16-expressing) PC cells were stably transfected with F114HA plasmids along with their vector only (CMV9) controls. Cell lysates were immunoblotted with indicated antibodies. (C) Immunofluorescence analysis of MiaPaCa-2 and T3M4 cells stably transfected with F114HA plasmids along with their vector only (CMV9) controls using anti-FLAG and anti-HA antibodies. DAPI was used to stain the nucleus. Scale bars, 10 μm. (D and E) Proliferation of MiaPaCa-2 (D) and T3M4 (E) cells was measured by the WST1 assay: control cells (black line) and F114HA expressing cells (grey line). Data represent mean ± s.e.m of a representative experiment (n=4, Student's *t*-test, *P<0.05, **P<0.001). Cell population doubling time (*T*_d_) was calculated from the growth rate during the exponential growth phase (day3 – day5) using the following formula: *T*_d_ = (0.693x*Δt*)/*ln*(*N*_t_/*N*_0_), where *Δt* is time interval between two stages of growth, *N*_t_ is cell density at time *t* and *N*_0_ is the cell density at initial time.

Although previous studies addressed the functional significance of various lengths of carboxyl-terminal MUC16 fragments (283 and 413 amino acids) in ovarian, breast and colon cancer cells, none demonstrated whether a cleaved MUC16 is generated following ectopic expression of these fragments [19,24,34]. Since the cleavage of MUC16 in the last (56^th^) SEA domain is predicted to be at ‘NFSPLARRVDR’ site that lies 50 residues upstream to the transmembrane domain in the last SEA domain [10], we reasoned that use of carboxyl-terminal 114 amino acids that includes the above mentioned cleavage site would be the smallest fragment that can generate the functional cell-associated MUC16. Due to lack of antibodies for the juxta-membrane region of MUC16, we generated a dual epitope-tagged mammalian expression construct using 114 carboxyl-terminal fragment of MUC16 with N-terminal FLAG-tag and a C-terminal HA-tag (Figure 1A). The resultant control (p3X-FLAG-CMV9 or CMV9) and MUC16-Cter (p3X-FLAG-114HA or F114HA) expression constructs were stably transfected into MUC16 non-expressing MiaPaCa-2 and expressing T3M4 PC cells. Expression of MUC16-Cter was verified by immunoblot and immunofluorescence analyses using anti-FLAG and anti-HA antibodies (Figures 1B and 1C). A unique ~17 kDa product representing the cleaved carboxyl-terminus of MUC16 was present in HA but not in FLAG-immunoblot (Figure 1B). Although we are not able to show cleavage of endogenous MUC16 owing to commercial unavailability of CTD specific antibody, Davies *et al*., [25] using an antibody against MUC16 CTD (developed in-house), demonstrated a similar ~17 kDa cleaved product in NHBE cells. This supports our findings in the ectopic expression system.

A study by Seelenmeyer C *et al.*, demonstrated that a C-terminal 1148 amino acid fragment of MUC16, despite lacking a putative N-terminal signal peptide is trafficked to the cell surface by the conventional secretory pathway (Endoplasmic reticulum-Golgi) [35]. This finding has also been extrapolated to be true for the full length MUC16 (22,152 amino acids) lacking a putative N- terminal signal peptide (based on the Uniprot database) [35]. Since we are expressing much smaller fragments, we compared the influence of the leader peptide on MUC16-Cter expression and localization. For this, we used a p3X-FLAG- CMV10 vector system, which lacks the preprotrypsin leader and compared it with the p3X-FLAG-CMV9. Sub cellular distribution of MUC16-Cter (F114HA) was assessed using CMV9 and CMV10 expression system by indirect immunofluorescence microscopy under non-permeabilized (NP) and permeabilized conditions in HeLa cells. Under non-permeabilized condition, MUC16 C-ter expressed from the CMV9 system was displayed on the cell surface, which was not observed when expressed from the CMV10 system, assessed using the N- terminal FLAG antibody (Supplemental Figures 1B and 1F). This suggests the requirement of a leader peptide for appropriate routing to cell surface. On the other hand, when HA- antibody was used neither the CMV9 nor the CMV10 system had any staining under NP condition (Supplemental Figures 1B and 1F). A similar study was conducted using permeabilized condition, where the distribution of the FLAG and HA- tagged products were significantly different in both systems. While CMV10 had mostly cytoplasmic distribution (Supplemental Figure 1H), the CMV9 system showed more punctate, membranous and nuclear distribution (Supplemental Figure 1D). Therefore, all our studies were conducted using the vector system with N-terminal leader peptide (p3X-FLAG-CMV9).

To investigate whether MUC16-Cter imparts any pro-tumorigenic functions to PC cells, *in vitro* proliferation was measured using WST1 assay. Both MiaPaca-2 and T3M4-F114HA cells exhibited a significant increase in the proliferative potential with a ~ 6 – 7 h reduction in the doubling time (Figure 1D and 1E, *P<0.05, **P<0.001) compared to the control (CMV9) cells.

### MUC16-Cter promotes G2/M block with apoptotic resistance, a property associated with cancer stem-like cells, in PC cells

**Figure 2:**
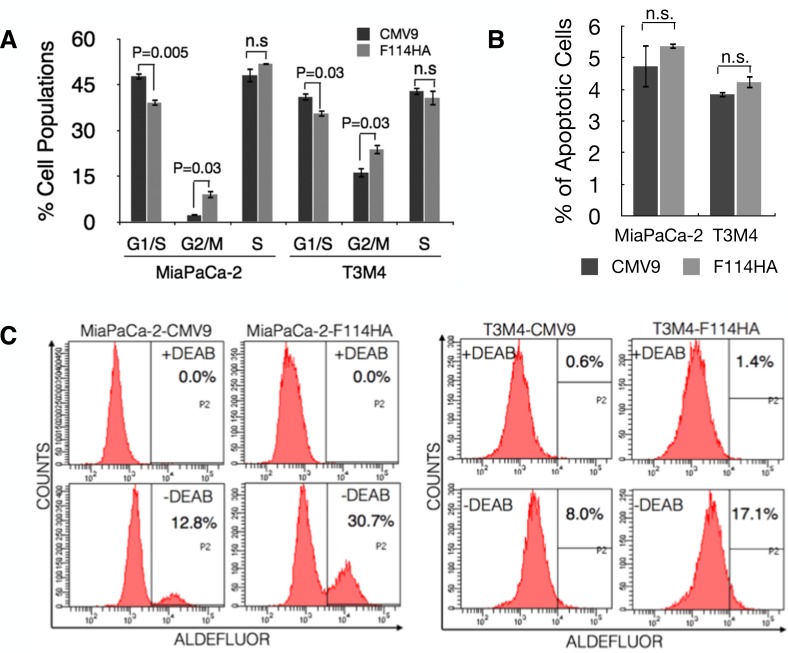
MUC16-Cter induces G2/M block in PC cells with apoptosis resistance (A) Cell cycle analyses were carried out in PC cells following a double thymidine block protocol. The percentage of cells in G1, S and G2/M phases of the cell cycle are of a representative experiment. Bars represent mean ± s.e.m, n=3. (B) The percentage of apoptotic cells was measured by Annexin-V and propidium iodide (PI) staining using FACS. Annexin-V^+^ and PI^−^ cells are considered to be apoptotic. Bars represent mean ± s.e.m, n=3. (C) Flow cytometric analysis of ALDH activity of PC cells were measured using ALDEFLUOR reagent in the presence or absence of ALDH1 inhibitor diethylamino-benzaldehyde (DEAB). This is a representative experiment in which the ALDH-positive cells measured by flow cytometry from cells treated with DEAB (top panel) were then applied to the untreated cells (bottom panel). The percentages of cells are indicated in the respective plots.

Previously MUC16 was shown to induce rapid G2/M transition in MDA-MB-231 breast cancer cells [23]. However, cell cycle analysis to gaze at the role of MUC16 C-ter in PC cells, resulted in significant accumulation of cells in the G2/M phase (Figure 2A, P=0.03) as opposed to rapid G2/M transition [23]. Interestingly, this was unaccompanied by an increase in apoptosis (Figure 2B), a property expected of cells blocked in the G2/M phase. Extended G2/M phase with increased resistance to apoptosis is a property commonly ascribed to cancer stem cells (CSCs) [36-38]. To examine whether ectopic expression of MUC16-Cter confers CSC phenotype in PC cells, ALDH activity was measured that is shown to be more relevant [39] and efficient [40] in identification of PC stem cells. Accordingly, we observed more than two-fold increase in the ALDH^+^ populations in cells expressing MUC16-Cter compared to control cells (Figure 2C).

### MUC16-Cter mediated up regulation of *LMO2* and *NANOG* is dependent on JAK2

**Figure 3:**
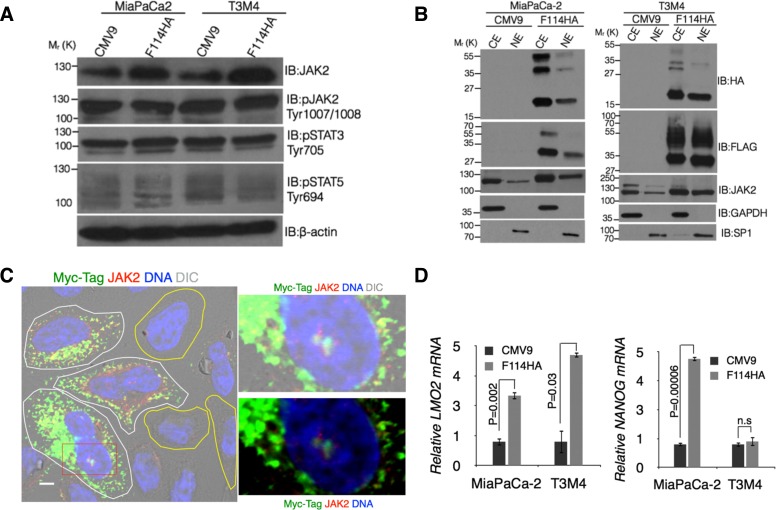
MUC16-Cter mediated increased nuclear JAK2 leads to up regulation of *LMO2* and *NANOG* (A) MUC16-Cter leads to increase in total JAK2. Whole cell lysates from control (CMV9) and MUC16-Cter expressing (F114HA) PC cells were immunoblotted with indicated antibodies. (B) Control and MUC16-Cter expressing PC cells were lysed and nuclear (NE) and cytosolic (CE) fractions were isolated. Western blots were performed on the subcellular fractions with indicated antibodies. (C) Immuno-colocalization of JAK2 and MUC16-Cter. HeLa cells transiently transfected with pSecTag2C-HA114Myc (i.e. HA114Myc) were analyzed for colocalization of MUC16-Cter (Myc-tag, anti-mouse antibody) and JAK2 (anti-rabbit antibody) by immunofluorescence microscopy. Transfected and untransfected cells were marked with white and yellow lines respectively. Scale bars, 5 μm. (D) Nuclear JAK2 leads to up regulation of *LMO2* and *NANOG*. Total RNA isolated from control and MUC16-Cter expressing cells was used as a template for synthesis of cDNA and expression of *LMO2* and *NANOG* were analyzed using real time RT-PCR. Data represent mean ± s.e.m, n=3.

**Figure 4:**
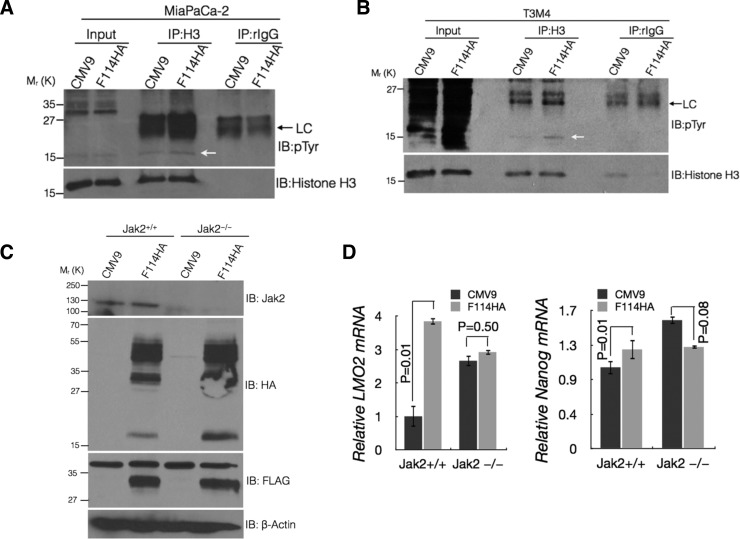
MUC16-Cter mediated up regulation of *LMO2* and *NANOG* is dependent on JAK2 (A and B) Enhanced tyrosine phosphorylation of Histone-H3 in cells expressing MUC16-Cter. Cell lysates from control and MUC16-Cter expressing MiaPaCa-2 (A) and T3M4 (B) PC cells were immunoprecipitated using anti-Histone-H3 or control (rabbit-IgG) antibodies and immunoblotted using indicated antibodies. Whole cell lysates from the respective cell types were used as input. (C) Mouse mammary cancer cells established from *MMTV-Neu;Jak2*_fl/fl_ mice with either pBabe-Puro (Jak2^+/+^) or pBabe-Puro-Cre (Jak2^−/−^) were transfected with control or F114HA plasmids. Cell lysates prepared from these cells were immunoblotted with indicated antibodies. (D) Dependence of MUC16-Cter on Jak2 in upregulating Lmo2 and _Nanog_. Mouse mammary cancer cells established from *MMTV-Neu;Jak2*^fl/fl^ mice with either pBabe-Puro (Jak2^+/+^) or pBabe-Puro-Cre (Jak2^−/−^) were transfected with control or F114HA plasmids. This was followed by total RNA extraction, cDNA synthesis and expression of *Lmo2* and *Nanog* using real time RT-PCR. Data represent mean ± s.e.m, n=3.

Having observed an accumulation of cells in the G2/M phase with apoptotic resistance, we sought to investigate the mechanism of MUC16-Cter mediated enrichment of ALDH^+^ population. For this we focused on JAK2, a non-receptor tyrosine kinase, for two reasons. First, JAK2 has been shown to interact with MUC16 possibly by its FERM domain [23]. Second, studies in hematopoietic [28] and embryonic stem cells [29] have demonstrated that nuclear JAK2 phosphorylates tyrosine 41 of histone H3 (H3Y41) in a STAT-independent manner, up regulating *LMO2* and *NANOG*. Both LMO2 and NANOG have been shown to induce stem cell-like features during carcinogenesis [30-32]. An increase in the total JAK2 protein with no alterations in its down-stream effectors (Figure 3A) was observed in both MiaPaCa-2 and T3M4 PC cells expressing MUC16-Cter. No change in JAK2 mRNA was observed (Supplemental Figure 2A). Next, nuclear JAK2 level was assessed using sub-cellular fractionation of control and F114HA transfected MiaPaCa-2 and T3M4 cells. An increased nuclear JAK2 was observed in response to MUC16-Cter expression (Figure 3B) suggesting its involvement in mediating the functions of MUC16-Cter. To circumvent the use of same source (rabbit) antibodies for JAK2 and HA-tag (MUC16-Cter) in immunofluorescence for colocalization analysis, we used a C-terminal Myc-tagged version of 114 amino acids of MUC16-Cter construct (HA114Myc) in pSecTag2C vector with Igκ leader peptide. HeLa cells transiently transfected with a C-terminal Myc-tagged version of MUC16-Cter showed increased total and nuclear JAK2 (cells outlined in white line) compared to cells that are untransfected (cells outlined in yellow line) (Figure 3C). In addition, both MUC16-Cter (using anti-mouse Myc-tag antibody) and JAK2 (anti-rabbit antibody) were colocalized in the nucleus (Figure 3C). In accordance with the increased nuclear JAK2, target genes such as *LMO2* and *NANOG* were up regulated in cells expressing MUC16-Cter compared to control cells (Figure 3D). A more pronounced effect of MUC16-Cter is observed in *LMO2* expression compared to *NANOG* in T3M4 (Figure 3D) and Jak2^+/+^ mammary tumor cells (Figure 4D). In addition, inhibition of JAK2 in K562 cells by TG101209 demonstrates a significant abrogation of LMO2 expression with no influence on NANOG expression (Supplemental Figure 2D). Taken together, all the above data suggests that MUC16-Cter mediated nuclear JAK2 exerts a more robust effect on *LMO2* expression compared to *NANOG* and could be one of the reasons for not observing a statistically significant *NANOG* expression in T3M4 cells (Figure 3D).

It has been shown that phosphorylation of tyrosine 41 of histone 3 (H3Y41) by JAK2 leads to interference with the binding of heterochromatin protein 1α (HP1α) on the promoters of *LMO2* and *NANOG* resulting in their up regulation. Due to lack of commercial antibody specific for phospho-H3Y41, we immunoprecipitated H3 from the control and MUC16-Cter expressing MiaPaCa-2 and T3M4 cells followed by immunoblotting with the general phosphotyrosine antibody. An increased tyrosine phosphorylation of histone H3 (pTyr-H3) was observed in cells expressing MUC16-Cter (Figure 4A and 4B) compared to control. To further demonstrate that up regulation of *LMO2* and *NANOG* by MUC16-Cter is indeed dependent on JAK2, MUC16-Cter (F114HA) was expressed in mouse mammary cancer cells with (*Jak2^+/+^*) and without *Jak2* (*Jak2^−/−^*) [41] (Figure 4C). Mouse mammary cancer cells with intact *Jak2* (*Jak2^+/+^*) resulted in up regulation of *Lmo2* and *Nanog* in MUC16-Cter dependent manner and this dependence was abrogated in cells genetically deleted for *Jak2* (*Jak2^−/−^*) (Figure 4D). Similar findings of up regulated *LMO2* and *NANOG* were observed in response to a JAK2 specific inhibitor TG101209 in MiaPaCa-2 cells and that was independent of MUC16-Cter expression (Supplemental Figures 2B and 2C). However, TG101209 resulted in significant down regulation of *LMO2* in K562 chronic myelogenous leukemia cell line (Supplemental Figure 2D) as reported previously [28]. Although the levels *LMO2* and *NANOG* are high in the absence of JAK2, we conclusively demonstrate the JAK2 dependence of MUC16-Cter in up regulating *LMO2* and *NANOG* in cells that express JAK2. While NANOG has been widely implicated in stemness [32] and early stage PC [31], LMO2 is not well characterized in PC with the exception of one study associating it with better prognosis [42]. Thus our study reveals MUC16-Cter-JAK2-LMO2/NANOG axis to be a novel pathway responsible for mediating enrichment of PC cancer stem-like cells.

### MUC16-Cter imparts chemo resistance properties to PC cells

**Figure 5:**
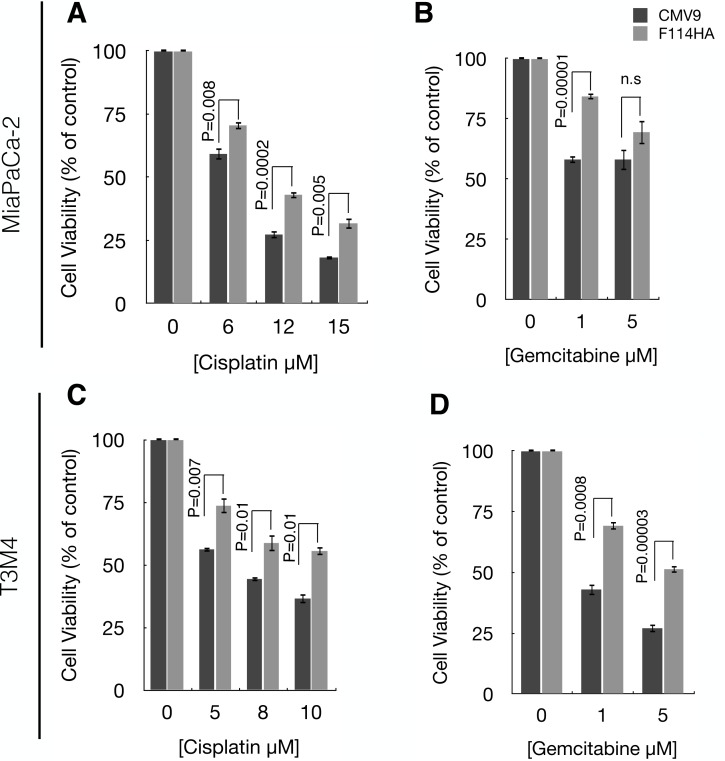
MUC16-Cter confers resistance to MiaPaCa-2 and T3M4 cells against gemcitabine and cisplatin Control (CMV9) and MUC16-Cter-expressing (F114HA) MiaPaCa-2 (A and B) and T3M4 (C and D) cells were either untreated (considered 100% viable or 0% cytotoxic) or treated with indicated concentration of cisplatin or gemcitabine. Forty-eight hours later, the viability of the cells was assessed by MTT assay and the bar graphs represent the mean ± s.e.m (n=4) percentage of cell death (cytotoxicity) compared to respective no drug controls.

Our previous results showed that MUC16-Cter leads to enrichment of ALDH^+^ CSCs. Accumulating evidence over the last decade has demonstrated the significance of CSCs in chemo resistance and metastasis in various cancers including PC [4-6]. Therefore, we assessed the chemo resistance properties of cells transfected with MUC16-Cter and observed a significant reduction in the cytotoxicity of both MiaPaCa-2 and T3M4 cells expressing MUC16-Cter compared to control in response to chemotherapeutic agents such as gemcitabine and cisplatin (Figures 5A-5D).

### Expression of MUC16-Cter leads to increased motility of PC cells

**Figure 6:**
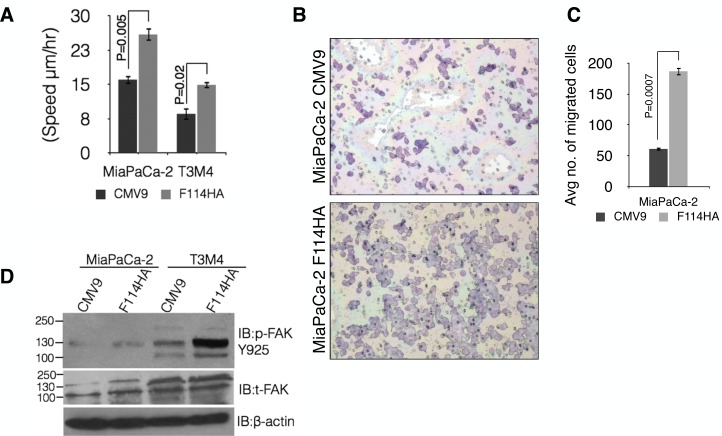
MUC16-Cter promotes motility of PC cells *in vitro* (A) Cell migration of control and F114HA transfected MiaPaCa-2 and T3M4 PC cells was monitored by live imaging of a scratch every 10 minutes interval for 48 hours in 1% serum-containing media with an Olympus IX81 motorized inverted microscope (Olympus America Inc., Center Valley, PA, USA) at three different spots. The speed of migration (mean ± s.e.m, n=3) was calculated by a ratio of distance migrated over time. (B and C) Control (CMV9) and MUC16-Cter (F114HA)-transfected MiaPaCa-2 cells were seeded onto filters with an 8 μm pore size in uncoated (Boyden's chamber) upper chambers in serum-free medium. The cells were allowed to migrate for 24 hours to the lower chamber having 10% FBS-containing medium as a chemo attractant. A representative image shows the motile cells in (B) that are quantitated in (C). Bars represent the average number of cells (mean ± s.e.m) per field (10 fields per chamber) and three chambers (n=3) per cell type. (D) Up regulation of FAK phosphorylation in cells transfected with MUC16-Cter compared to control cells. Whole cell lysates prepared from control (CMV9) and MUC16-Cter-expressing (F114HA) MiaPaCa-2 and T3M4 cells were immunoblotted with the indicated antibodies.

Approximately 85–95% of PC patients are diagnosed with either locally advanced or metastatic disease [43-45] and a previous study from our lab demonstrated that MUC16 expression is much stronger in metastatic lesions compared to the primary pancreatic tumor [33]. Therefore, we investigated the role of MUC16-Cter in PC cell motility using live imaging of scratch assay (Figure 6A) as well as uncoated porous membranes of 8 μm pore diameter (Boyden chamber). The speed of migration was found to be significantly higher in both MiaPaCa-2 (P=0.005) and T3M4 (P=0.02) cells expressing MUC16-Cter compared to control (Figure 6A). Similar observation was made in MiaPaCa-2-F114HA cells (P=0.0007) compared to control using Boyden chamber assay (Figures 6B and 6C). We did not observe any migration of T3M4 cells using Boyden chamber assay. In agreement with the increased motility, increased phosphorylation of focal adhesion kinase (FAK) was observed in cells with MUC16-Cter (Figure 6D) compared to control cells.

### MUC16-Cter promotes tumorigenic and metastatic properties of PC cells *in vivo*

**Figure 7:**
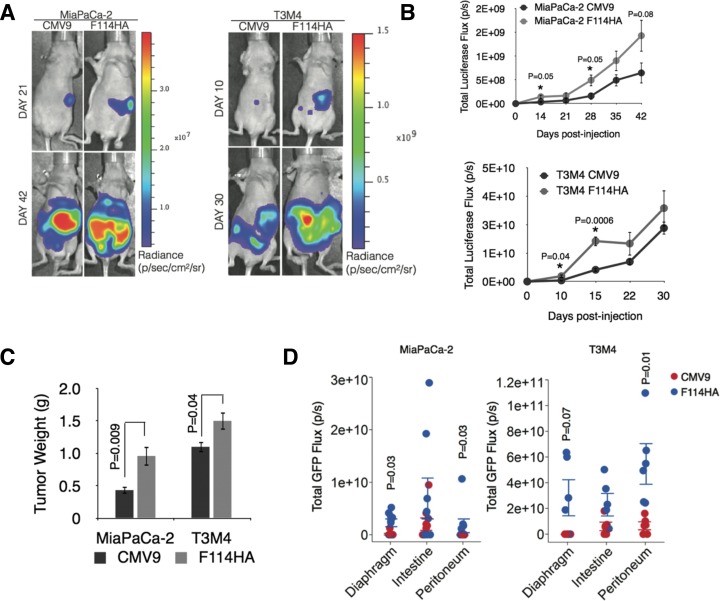
MUC16-Cter confers enhanced tumorigenic and metastatic properties to PC cells *in vivo* (A) Representative images are shown of nude mice whose pancreas were orthotopically implanted with 3×10^6^ MiaPaCa-2-Luc-eGFP (control, CMV9 and MUC16-Cter-F114HA transfected) or 0.25×10^6^ T3M4-Luc-eGFP (CMV9 and F114HA) cells respectively. Bioluminescence imaging performed on the indicated days shown. (B) Growth of primary and metastatic tumors were monitored by *in vivo* bioluminescence for the indicated days post-transplantation and are presented as mean ± s.e.m, (n=8 for MiaPaCa-2 and n=5 for T3M4 cells) per condition. Statistically significant bioluminescence was observed for MUC16 Cter-transfected compared to control cells on days 14 and 28 for MiaPaCa-2 (n=8) and days 10 and 15 for T3M4 (n=5) cells (Student's *t*-test, data were not adjusted for multiple comparison) (C) Increased primary tumor size at sacrifice in mice injected with F114HA transfected cells for both MiapaCa-2 and T3M4 cells. Bars represent mean ± s.e.m (Student's *t*-test, n=8 for MiaPaCa-2 and n=5 for T3M4). (D) The extent of metastasis to various organs was quantified as the total GFP flux (p/s). Means and s.e.m data are derived from one experiment of eight (MiaPaCa-2) and five (T3M4) mice per condition. Statistical significance was calculated using the Wilcoxon rank sum test.

We demonstrated that MUC16-Cter imparts proliferative, invasive and chemo resistance properties to PC cancer cells *in vitro* in addition to enrichment of ALDH^+^ CSCs. Next, we sought to investigate the *in vivo* relevance of MUC16-Cter with respect to tumorigenesis and metastasis using an orthotopic model of PC. To track the development and metastasis of PC growth *in vivo*, control and MUC16-Cter expressing MiaPaCa-2 and T3M4 cells were infected with lentivirus expressing luciferase-eGFP before being implanted into the pancreas of nude mice. For this experiment, 3 × 10^6^ MiaPaCa-2-Luc-eGFP (CMV9 and F114HA, n=8 per group) and 2.5 × 10^5^ T3M4-Luc-eGFP (CMV9 and F114HA, n=5 per group) were implanted in to the head of the pancreas and were monitored weekly for tumor growth and metastasis using *in vivo* bioluminescent imaging (Figure 7A). A significantly higher tumor growth and metastasis were observed in mice implanted with cells expressing MUC16-Cter as measured by the total luciferase flux (photons/sec) (Figure 7B). The animals implanted with MiaPaCa-2 and T3M4 cells were sacrificed at day 42 and 30 respectively. During autopsy of these mice, weights of the primary tumors were measured and were found to be significantly higher for both MiaPaCa-2 (P=0.009) and T3M4 (P=0.04) cells expressing MUC16-Cter (Figure 7C). Significant metastatic burden was observed in the diaphragm and peritoneum of mice implanted with cells expressing MUC16-Cter, which was quantified as the total GFP flux (photons/sec) using *ex vivo* GFP imaging of various organs (Figure 7D and Supplementary Figures 3A and 3B). In particular, peritoneal metastasis was significantly higher and was macroscopically visible to the naked eye (Supplemental Figure 3A). Histologically expression of HA-tagged MUC16-Cter was confirmed both at the primary and metastatic sites, demonstrating that the expression of MUC16-Cter was maintained all through the process of formation of primary and metastatic PC *in vivo* (Supplemental Figures 3C and 3D). Taken together, we demonstrate for the first time generation of a ~17 kDa cleaved MUC16 that is capable of promoting tumorigenic and metastatic spread of PC cells *in vivo*.

## DISCUSSION

MUC16 was initially identified as CA125 and is the most widely used serum biomarker particularly for recurrent OC following chemotherapy [46,47]. Recently, it was shown to be over expressed in multiple cancers including PC [17,18,33,48] and was flagged as the third most frequently mutated gene across tumor types [49]. Carboxyl-termini of transmembrane mucins generated following cleavage are hypothesized to be the business end of these proteins in multiple malignancies, but is shown only for MUC1. Although MUC16 is predicted to undergo proteolytic cleavage in the last and/or penultimate SEA domains close to the transmembrane domain [10,13], its precise cleavage has not been experimentally validated. A more detailed study addressing MUC16 cleavage shows that the cleavage is distinct from the predicted sites (Das et al., submitted for publication elsewhere). Therefore, one of the most important findings of the current study is the demonstration of a ~17 kDa cleaved MUC16 in PC cells using a dual-epitope tagged 114 carboxyl-terminal amino acids (F114HA), which was not shown by previous studies addressing the role of carboxyl-terminal MUC16 [19,20,24,34]. Support towards the endogenous existence of such ~17 kDa fragment comes from a study using in-house antibody for the CTD of MUC16 in NHBE cells [25].

Cells blocked at the G2/M phase of the cell cycle with apoptosis resistance are shown to possess features of CSCs with increased proliferative capacity [36-38] in multiple tumors. Similar characteristics of G2/M accumulation with apoptosis resistance and increased proliferative potential were observed in MiaPaCa-2 and T3M4 cells ectopically expressed with F114HA, that generates a functional cleaved MUC16. Further, our assessment of CSCs using ALDH activity, which is shown to be more relevant [39] and efficient [40] in identifying the PC tumor initiating cells compared to CD133, showed MUC16-Cter mediated enrichment of ALDH^+^ CSCs. The enrichment of ALDH^+^ CSCs suggests MUC16/CA125 may not be just a passive biomarker in recurrent metastatic OC [50] but could be actively involved in the recurrence by inducing CSC like features. As early-disseminated CPCs with CSC like features are considered to the primary mode of establishing metastatic PC [2], an increased expression of MUC16 in metastatic PC compared to primary tumors [33] suggests the role of MUC16-Cter to be critical in this process. Accordingly, we observed significantly higher tumorigenic and metastatic ability of cells expressing MUC16-Cter *in vitro* and *in vivo* compared to control. Of all the metastatic sites, MUC16-Cter mediated peritoneal metastasis in MUC16 non-expressing MiaPaCa-2 cells was interesting because, our previous study [51] using ectopic expression of MUC4 in these cells (MUC4 is not expressed in MiaPaCa-2) primarily resulted in liver metastasis. This is important from two aspects. First, this demonstrates that different mucins exhibit different metastatic tissue tropism and therefore explains the requirement of expression of more than one mucin in the same patient. Second, MUC16 is a critical mediator of peritoneal metastasis of OC cells by its interaction with mesothelin [52]. Therefore, MUC16-Cter mediated enhanced peritoneal metastasis in PC is probably reminiscent of its role in OC. However, further studies will be required to elucidate its exact molecular mechanism in PC.

Another important aspect of CSC is imparting chemoresistance. A previous study [20] using a 283aa long MUC16-Cter showed increased resistance of SKOV3 cells to cisplatin, however the mechanism was unknown. Here, we provide evidence that MUC16-Cter leads to enrichment of ALDH^+^ CSCs that is at least in part provides resistance to gemcitabine and cisplatin in PC cells.

Mechanistically, we demonstrated that MUC16-Cter results in nuclear translocation of JAK2, which preferentially phosphorylates histone-3 and up regulates stemness-specific genes such as *LMO2* and *NANOG*, which was further validated using Jak2 deficient (*Jak2^−/−^*) mouse mammary tumor cells. However, higher basal levels of *LMO2*/*Lmo2* and *NANOG*/*Nanog* in cells with JAK2 inhibition or Jak2 deletion suggests multiple factors influencing the expression of *LMO2*/*Lmo2* and *NANOG*/*Nanog*. On the other hand, K562 cells showed expected down regulation of *LMO2* upon JAK2 inhibition. The effectiveness of JAK2 inhibition in K562 cells, but not in epithelial tumor cells is probably one of the reasons why JAK2 inhibitors are more widely used in clinical trials of hematological malignancies but not of the epithelial solid tumors.

Our basic understanding of the functional and mechanistic involvement of MUC1-Cter in tumorigenesis resulted in development of peptide inhibitors such as GO-203-2C, which is under phase-I clinical trial (NCT01279603) against patients with advanced solid tumors and lymphoma. Here, we showed generation of a functional cleaved MUC16 that imparts tumorigenic, metastatic and drug resistant properties to PC cells partly by enrichment of ALDH^+^ CSCs, which in turn is dependent on nuclear JAK2 mediated up regulation of stemness specific genes *LMO2* and *NANOG*. Therefore, it is conceivable that therapeutic strategies that can be targeted against MUC16-Cter will be critical in treating MUC16 expressing pancreatic cancer patients. We propose that this can be achieved in two different ways.

First, preventing cleavage of MUC16 will lead to reduced nuclear translocation (Das et al., submitted for publication elsewhere) and therefore have reduced influence on gene expression. In this context, decreased levels of LMO2 and NANOG leads to reduction in the ALDH^+^ pancreatic CSCs. Besides, reduced cleavage will lead to increased surface representation of MUC16 and therefore may enhance the efficacy of the CA125 antibody based therapeutics such as Oregovomab and Abagovomab [16]. Our study demonstrates that brefeldin A (BFA) that induces a rapid and reversible block in the secretory pathway, prevents the cleavage of MUC16 (Das et al., submitted for publication elsewhere). Therefore, it is tempting to speculate the use of Breflate (a prodrug form of brefeldin-A, NSC656202) [53] to prevent cleavage of MUC16 and its associated tumorigenic functions as an interesting therapeutic avenue owing to the increased reliance of tumor cells on the secretory pathway than their normal counterparts [53,54].

Second, antibody based therapeutics using antibodies against the juxta-membrane ectodomain and/or cytoplasmic domain of MUC16 may prove to be more effective than CA125 antibody based therapeutics such as Oregovomab and Abagovomab that are met with very limited success [16]. One of the reasons for the failure of these antibodies is binding of these antibodies to circulating (shed) MUC16 i.e CA125, reducing the drug delivery to target cells [16]. This can be overcome by using antibody-based therapeutics against the carboxyl-terminal MUC16. Therefore, further basic understanding of the mechanism(s) of MUC16 cleavage, nuclear translocation and biology of MUC16-Cter in addition to development of novel antibodies that can effectively bind to the cell associated MUC16-Cter would be critical in devising successful mucin based therapeutic strategies against multiple tumor types including PC.

## MATERIALS AND METHODS

### Cell culture and transfections

HeLa and pancreatic cancer cells MiaPaCa-2 and T3M4 were grown in DMEM with 10% heat-inactivated FBS/antibiotics. MiaPaCa-2 cells were established from the tumor in pancreas of a Caucasian male and harbors the most commonly found *KRAS^G12D^* oncogenic mutations observed in PC patients. Phenotypically, these cells are large with abundant cytoplasm, display aneuploidy and possess a tendency to grow in multiple layers [55,56]. T3M4 cells were established from a primary exocrine pancreatic carcinoma of a Japanese male and were also found to harbor *KRAS^G61H^* oncogenic mutations. These cells grow in a monolayer sheet with epithelial morphology resembling that of the original tumor [57,58]. K562 cells (kind gift from Dr. Javed Iqbal, UNMC) were grown in suspension culture in RPMI-medium with 10% FBS/antibiotics. *MMTV-Neu; Jak2^fl/fl^* cells transfected with either pBabe-Puro or pBabe-Puro-Cre were grown in DMEM/F12 medium with 10% heat-inactivated FBS/antibiotics and 7 μg/ml puromycin [41]. MiaPaCa-2 and T3M4, HeLa and *MMTV-Neu;Jak2^fl/fl^* cells were transfected using Lipofectamine 2000 (Invitrogen) according to the manufacturer's instructions. MiaPaCa-2 and T3M4 cells were selected for G418 (400-600 μg/ml) resistance to generate the stable cells. These stable cells were maintained in 400 μg/ml G418 only except during the experimental procedure. Both MiaPaCa-2 and T3M4 cells transfected with control (CMV9) and MUC16-Cter (F114HA) plasmids were stably transduced with lentivirus expressing firefly luciferase and eGFP (GeneCopoeia) for monitoring tumor growth and metastasis *in vivo*.

### Plasmids and cloning strategy

Plasmids and constructs used in this study were generated for the purpose of studying MUC16 cleavage and is submitted for publication elsewhere (Das et al., submitted for publication elsewhere). Briefly, standard PCR and molecular cloning techniques were used to make constructs. For expression in the mammalian system, p3X-FLAG-CMV9 and p3X-FLAG-CMV10 (Sigma-Aldrich) (termed as F114HA) and pSecTag2C (Invitrogen – Life Technologies) (termed as HA114Myc) plasmids were used where DNA fragments encoding the carboxyl-terminal region of MUC16 (114 amino acids) was amplified by reverse transcriptase PCR (RT-PCR) and was cloned into the respective expression vectors. The primers used in the generation of the constructs are outlined in Supplemental Table-1. All the constructs were verified by sequencing.

### Immunoprecipitation

For immunoprecipitation, cells were lysed in IP buffer (50 mM Tris-HCl pH-7.4, 300 mM NaCl, 5 mM EDTA, 1% NP-40) containing complete protease inhibitor cocktail, 2 mM Na_3_VO_4_, 10 mM NaF and 1 mM PMSF on ice for 30 minutes. Cell lysates were clarified by centrifugation and were immunoprecipitaed with indicated antibodies overnight at 4° C. Protein complexes were isolated by incubation with Protein-A, Protein-G or Protein-A/G Agarose beads (Santa Cruz Biotechnology) for 2-4 h. Immunoprecipitates were washed 3-5 times with IP buffer, boiled with SDS sample buffer and analysed by immunoblotting as described below using indicated antibodies.

### Immunoblotting

Cells were lysed with radioimmunoprecipitation buffer (50 mM Tris-HCl pH-7.5, 150 mM NaCl, 1% NP-40, 0.5% sodium deoxycholate, and 0.1% SDS) supplemented with protease inhibitor mixture, 2 mM Na_3_VO_4_, 10 mM NaF and 1 mM PMSF on ice. Cell lysates were cleared by centrifugation and quantified using the bicinchoninic acid method. Proteins (10-40 μg) were separated by SDS–PAGE under reducing conditions and blotted onto a PVDF membrane (Millipore). Membranes were probed with specific antibodies. Blots were washed and probed with respective secondary peroxidase-conjugated antibodies, and the bands visualized by chemiluminescence. The following antibodies were used: mouse monoclonal antibodies for FLAG-Tag (1:3000, Sigma), β-Actin (1:5000, Sigma), phospho-Tyrosine (1:1000, Cell Signaling), t-FAK (1:200, Santacruz Biotech), rabbit monoclonal antibodies for HA-Tag (1:2000, Cell Signaling), JAK2 (1:1000, Cell Signaling), pJAK2-Y1007/1008 (1:1000, Cell Signaling), pSTAT3-Y705 (1:1000, Cell Signaling), pSTAT5-Y694 (1:1000, Cell Signaling), GAPDH (1:1000, Cell Signaling), SP1 (1:1000, Cell Signaling), pFAK-Y925 (1:1000, Cell Signaling), rabbit polyclonal antibodies for Histone H3 (1:1000, Abcam).

### Subcellular fractionation

Subcellular fractionations were carried out using subcellular protein fractionation kits (Thermo Scientific and G-Biosciences) according to the manufacturers' instructions.

### *In vitro* assays of cell migration

The migratory potential of MiaPaCa-2 and T3M4 cells was tested using live imaging of wound-healing assay. To monitor migration rate scratches were made in confluent cell cultures grown in 6-well plates and were placed into a live cell-imaging incubator (37° C, 5% CO_2_), incubated for 48 h in DMEM+1% FBS. Cells were visualized with an Olympus IX81 motorized inverted microscope (Olympus America Inc) and the images were analyzed with Slidebook version 5.5 (Intelligent Imaging Innovations). Speed of migration was calculated by a ratio of distance travelled (μm) over time (h) taken at three different positions for each well. In addition, the migratory potential of MiaPaCa-2 cells was also tested using 6-well inserts of 8 μm pore size (BD Biosciences) as described previously [51].

### *In vivo* tumorigenesis and metastasis assay

Six-week-old nude mice were anaesthetized by intraperitoneal injection of xylazine (10 mg/kg) and ketamine (100 mg/kg). A left lateral laparotomy was performed on the abdomen and MiaPaCa-2 (3×10^6^) and T3M4 (2.5×10^5^) cells suspended in 50 μl of PBS were injected into the head of the pancreas. *In vivo* bioluminescent imaging was performed on indicated days using Xenogen IVIS-100 (Xenogen) following intraperitoneal injections of 100 μl D-luciferin (150 mg/kg) under isoflurane anaesthesia. After 42 (for MiaPaCa-2 cells) or 30 days (for T3M4 cells) mice were sacrificed, metastasis to distant organs was evaluated by *ex vivo* GFP imaging using Xenogen IVIS-100, weight of the primary tumors was documented. Total bioluminescence photon flux (photons/second) and total GFP fluorescence photon flux (photons/second) were analyzed by region of interest measurements in Living Image 4.4 (Perkin Elmer). These raw data were normalized to controls. University of Nebraska Medical Center Institutional Animal Care and Use Committee approved the procedures for all experiments performed with mice.

### *In vitro* cell proliferation assay

Cell proliferation was measured by the WST-1 assay according to the manufacturer's instructions as described previously [59].

### Cell cycle analysis

Double thymidine block was carried out to study cell cycle distribution as described previously [23].

### Annexin-V staining and flow cytometry

Early and late apoptotic cells were detected using annexin-V-FLUOS staining kit (Roche) as described previously [23].

### Cytotoxicity assay using MTT

Cytotoxicity to gemcitabine and cisplatin was determined using the MTT assay as described previously [59].

### Aldefluor assay

ALDH activity was determined using the ALDEFLUOR assay (StemCell Technologies) according to the manufacturer's instructions. Briefly, cells were trypsinized, washed twice with PBS, resuspended in Aldefluor assay buffer (ASB) and incubated with activated ALDH substrate (BAAA). For negative control, an aliquot of treated cells was incubated with an ALDH inhibitor DEAB. Cells were incubated at 37°C for 30-45 min, centrifuged for 5 min and the pellet was resuspended in 500μl of ASB with propidium iodide and passed through a 30 μm filter and were analyzed by flow cytometry. The ALDH positive gate was created based on DEAB-treated cells stained with Aldefluor.

### Immunofluorescence microscopy

HeLa cells grown on cover slips were transiently transfected with pSecTag2C-HA-MUC16-114-Myc (i.e. HA114Myc) plasmids using Lipofectamine (Invitrogen). HeLa cells grown on cover slips were transiently transfected with p3X-FLAG-CMV9 (CMV9), p3X-FLAG-CMV9-114HA (CMV9-F114HA), p3X-FLAG-CMV10 (CMV10) and p3X-FLAG-CMV10-114HA (CMV10-F114HA) using Lipofectamine. MiaPaCa-2 and T3M4 cells stably transfected with p3X-FLAG-CMV9 and p3X-FLAG-114HA were grown on cover slips for 24 hours. Then the cells were washed twice with PBS and fixed with 4% paraformaldehyde in PBS (pH-7.4) for 10 min. After washing with PBS, cells were quenched with 30 mM glycine. Cells were then either non-permeabilized or permeabilized with 0.1% Triton-X-100 for 10 min and blocked with 10% normal goat serum (NGS) in PBS for 1 h. Cells were incubated with appropriate antibodies (anti-HA; 1:300, anti-FLAG; 1:500, anti-Myc; 1:300, anti-JAK2; 1:300) for 1 h in PBS containing 2% NGS. Cells were washed with PBST (x3) and PBS (x1) and incubated with Alexa Fluor 488-conjugated donkey anti-mouse and Alexa Fluor 568-conjugated donkey anti-rabbit (Life Technologies) antibodies for 30 min. The cells were washed with PBST (x3) and PBS (x1) and mounted in Vectashield with DAPI (Vector Laboratories).

### Immunohistochemical staining of orthotopic tumors

Formalin-fixed mouse tissues were embedded in paraffin, sectioned (5 μm) and stained with hematoxylin and eosin and with anti-HA antibodiy (1:300). Standard procedure was followed for immunohistochemical analysis [60].

### qPCR analysis

RNA extraction and qPCR was performed as described previously [61]. Primers used in the study are listed in Supplemental Table-2.

### Statistics

Results are expressed as mean ± s.e.m. Statistical analyses were carried out using Student's *t*-test, unless otherwise mentioned.

## SUPPLEMENTARY MATERIAL, FIGURES, TABLES



## Abbreviations

C-Ter: carboxy-terminal
CSC: cancer stem cell
SEA: sperm protein, enterokinase, agrin
JAK2: janus kinase 2
