# Supplementary material for: Carboxyl-terminal domain of MUC16 imparts tumorigenic and metastatic functions through nuclear translocation of JAK2 to pancreatic cancer cells
Source: Oncotarget. 2015 Jan 21;6(8):5772–87. doi: 10.18632/oncotarget.3308 (PMC4467401; doi:10.18632/oncotarget.3308)
Supplement: Supplementary file 1 [file oncotarget-06-5772-s001.pdf]

# Carboxyl-terminal domain of MUC16 imparts tumorigenic and metastatic functions through nuclear translocation of JAK2 to pancreatic cancer cells

**Supplementary Material**

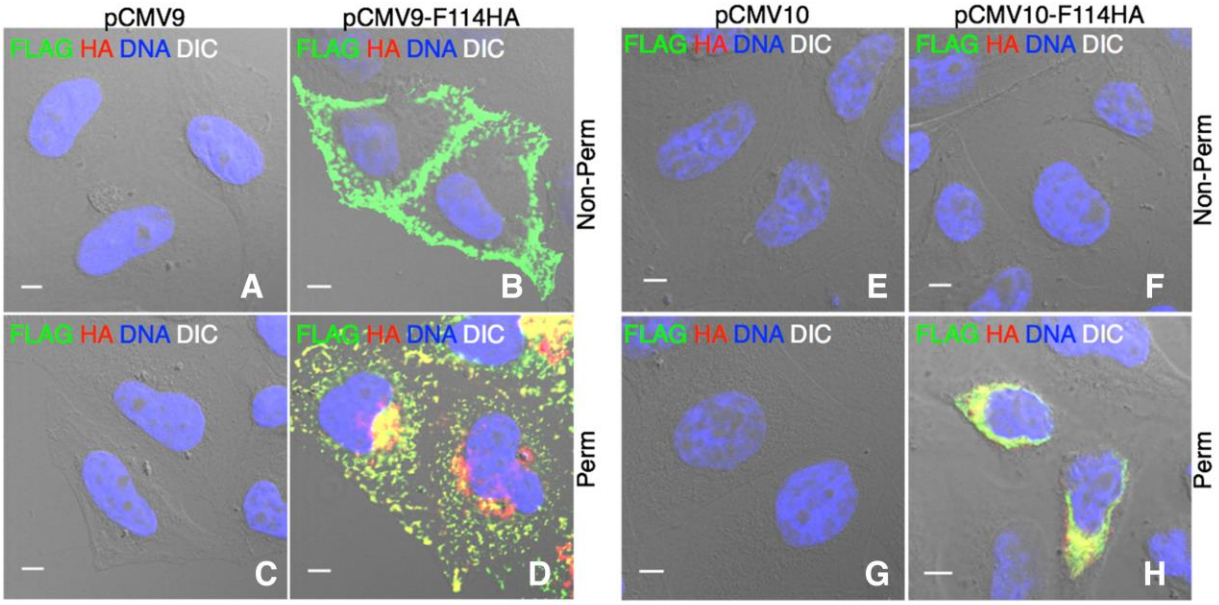


**Supplemental Figure 1. Requirement of N-terminal signal peptide for appropriate membrane targeting of carboxyl-terminal portion of MUC16.** HeLa cells transiently transfected with CMV9-F114HA (left panel, **B** and **D**) and CMV10-F114HA (right panel, **F** and **H**) constructs along with their respective controls (CMV9: left panel, **A** and **C**; CMV10: right panel, **E** and **G**) were analyzed for the surface and intracellular localization of both FLAG and HA-tagged portions of MUC16-Cter under non- permeabilizing (Non-Perm) and permeabilizing (Perm) conditions using immunofluorescence microscopy. Cells were analyzed for the surface an intracellular localization of both FLAG (green) and HA-tagged (red) portions of MUC16-Cter. DAPI was used to stain the nucleus (DNA). Scale bars, 5μm.

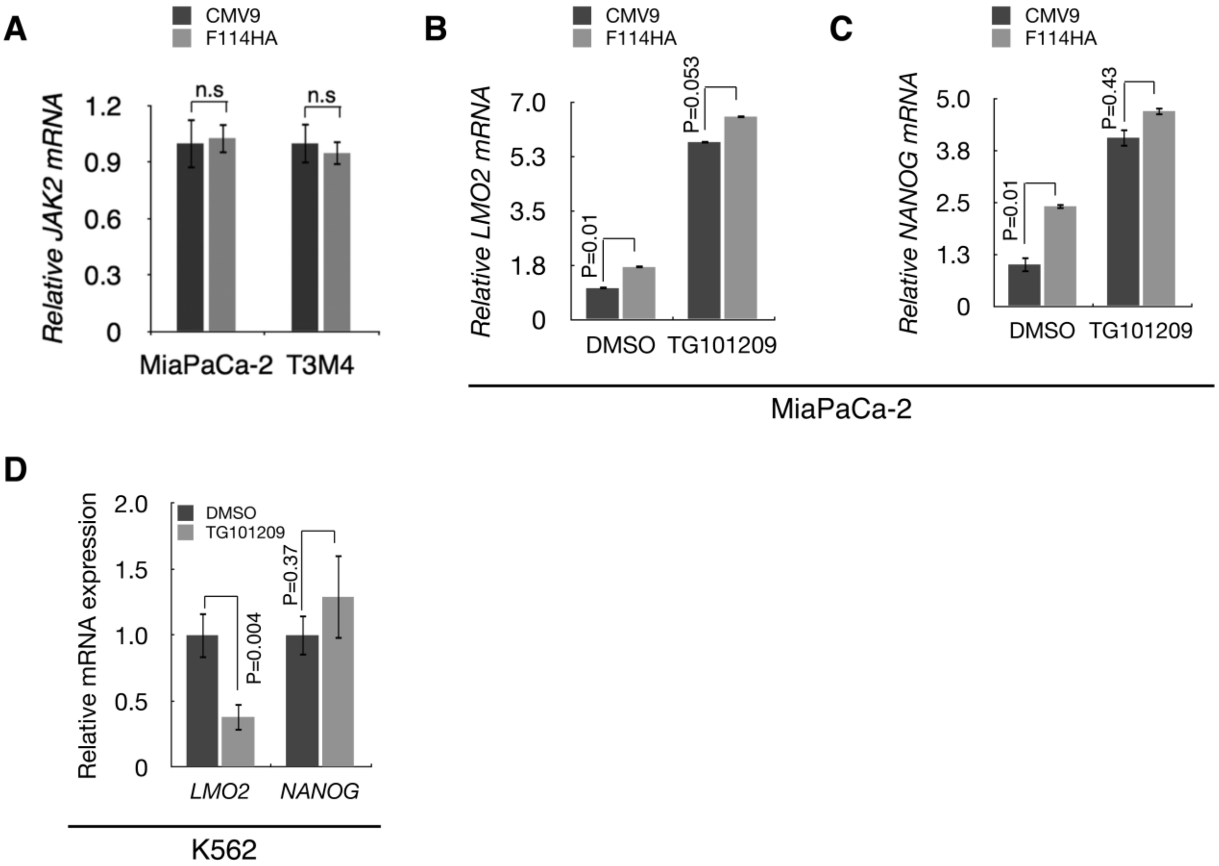


**Supplemental Figure 2. MUC16-Cter does not up regulate *JAK2* at the mRNA level and inhibition of JAK2 in MiaPaCa-2 cells leads to up regulation of basal level of *LMO2* and *NANOG* independent of MUC16-Cter.** (**A**) Quantitative real-time PCR analysis of *JAK2* expression from control and MUC16-Cter expressing cells. Data represent mean ± s.e.m, n=3. (**B** and **C**) MiaPaCa-2 cells stably transfected with either control (CMV9) or MUC16-Cter (F114HA) were treated with either DMSO or TG101209 (3 µM) for 3 h. Expression of *LMO2* and *NANOG* was analyzed using real-time PCR. (**D**) K562 cells were treated with either DMSO or TG101209 for 3 h. Expression of *LMO2* and *NANOG* was analyzed using real-time PCR.

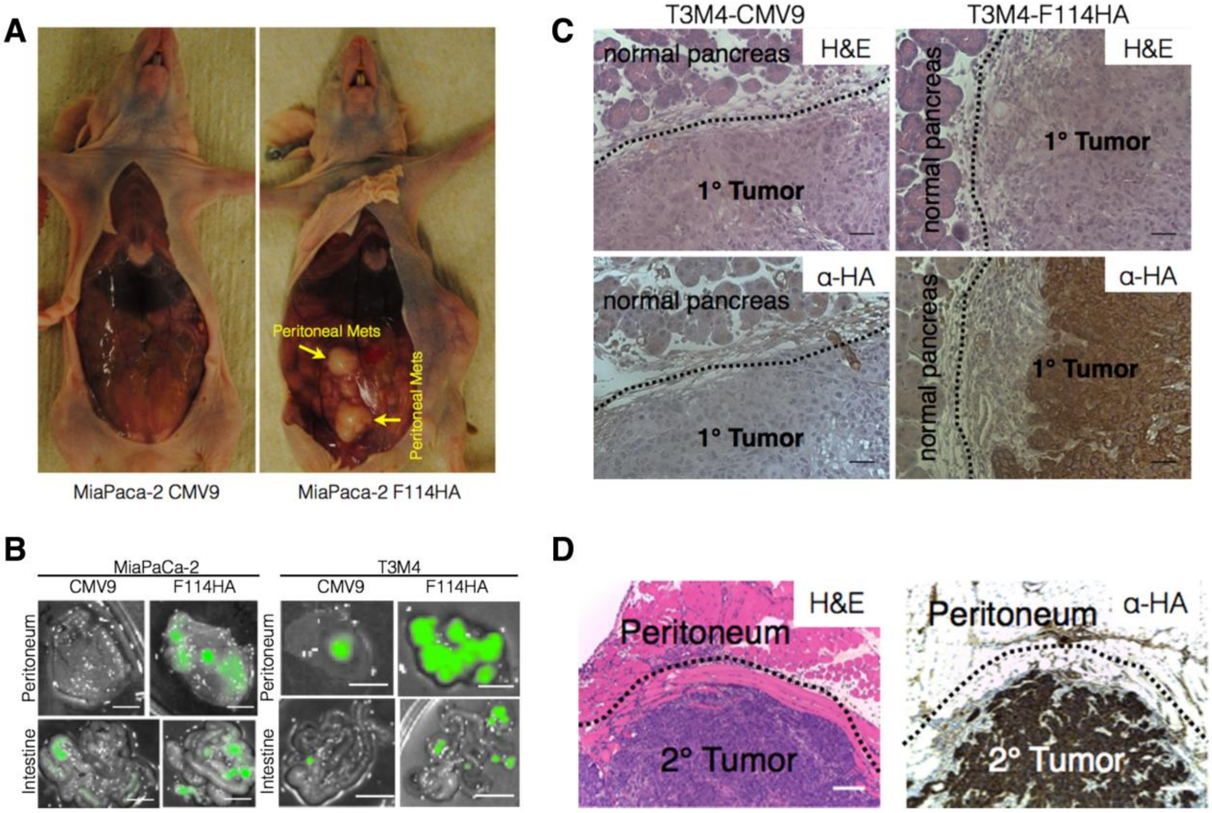


**Supplemental Figure 3. MUC16-Cter imparts metastatic phenotypes PC cells.** (**A**) Representative photographs of mice receiving MUC16-Cter expressing MiaPaCa-2 cells showing extensive peritoneal metastasis (yellow arrows), which was not observed in the mice receiving control cells. (**B**) *Ex vivo* GFP fluorescence images of representative metastatic sites (peritoneum and intestine) demonstrate the enhanced metastatic capability of MUC16-Cter-transfected MiaPaCa-2 and T3M4 cells over the control cells. Scale bars, 10 mm. (**C**) Sections of primary tumors from mice injected with control and MUC16-Cter-expressing cells were stained with haematoxylin and eosin (H&E, top panel) or stained with anti-HA antibodies (α-HA, bottom panel). Scale bars, 500 μm. (**D**) Sections of metastatic tumor from mice injected with MUC16-Cter-expressing T3m4 cells were stained with haematoxylin and eosin (H&E, left) or stained with anti-HA antibody (α-HA, right). Scale bars, 500 μm.

**Supplemental Table-1** Primers used to generate the constructs (Restriction Sites are underlined)

**F114HA** (in p3X-FLAG-CMV9 and p3X-FLAG-CMV10 vector)

Forward Primer: 5’−CTGAAGATCTAACTTCTCGCCACTGGCTCGGAGAG−3’

Reverse Primer:
5’−TACGGGATCCTCAAGCGTAATCTGGAACATCGTATGGGTAACCACCTTGCAGAT
CCTCCAGGTCTAGGTG−3’

**HA114Myc** (in pSecTag2C vector)

Forward Primer:
5’−CTTAAAGCTTTATACCCATACGATGTTCCAGATTACGCTTTCATCAATGGCTATG
CAC−3’

Reverse Primer:
5’−TACGCTCGAGTTGCAGATCCTCCAGGTCTAGGTGTGACTGGTAGTAGCCTGGG
CAC−3’

**Supplemental Table-2.** Primers used in the study (for Real-Time PCR)

LMO2 Forward primer: 5’- CGGCGCCTCTACTACAAACT-3’

LMO2 Reverse primer: 5’-GAATCCGCTTGTCACAGGAT-3’

NANOG Forward primer: 5’-GAAACAGAAGACCAGAACTGTG-3’

NANOG Reverse primer: 5’- GGTCTTCACCTGTTTGTAGC-3’

JAK2 Forward primer: 5’-GTCCACCAGCGGAATTTATGCG-3’

JAK2 Reverse primer: 5’-CATATAGATCTCATCTGGGCATCC-3’

β-ACTIN Forward primer: 5’-TGGACATCCGCAAAGACCTG-3’

β-ACTIN Reverse primer: 5’-TGGACATCCGCAAAGACCTG-3’

Lmo2 Forward Primer: 5’-CAGCAGAACATAGGGGACCG-3’

Lmo2 Reverse Primer: 5’-TCCTGACCAAAAAGCCTGAGAT-3’

Nanog Forward Primer: 5’-TACCTCAGCCTCCAGCAGAT-3’

Nanog Reverse Primer: 5’-GGTGCTGAGCCCTTCTGAAT-3’
